# Brain network topology predicts participant adherence to mental training programs

**DOI:** 10.1162/netn_a_00136

**Published:** 2020-07-01

**Authors:** Marzie Saghayi, Jonathan Greenberg, Christopher O’Grady, Farshid Varno, Muhammad Ali Hashmi, Bethany Bracken, Stan Matwin, Sara W. Lazar, Javeria Ali Hashmi

**Affiliations:** Department of Anesthesia, Pain Management, and Perioperative Medicine, Dalhousie University, NSHA, Halifax, Canada; Harvard Medical School, Mass General Hospital, Boston, MA, USA; Department of Anesthesia, Pain Management, and Perioperative Medicine, Dalhousie University, NSHA, Halifax, Canada; Faculty of Computer Science, Dalhousie University, Halifax, Canada; Massachusetts Institute of Technology, Cambridge, MA, USA; Charles River Analytics, Cambridge, MA, USA; Faculty of Computer Science, Dalhousie University, Halifax, Canada; Institute of Computer Science, Polish Academy of Sciences, Warsaw, Poland; Harvard Medical School, Mass General Hospital, Boston, MA, USA; Department of Anesthesia, Pain Management, and Perioperative Medicine, Dalhousie University, NSHA, Halifax, Canada

**Keywords:** Resting-state fMRI, Mental training programs, Machine learning, Graph theory, Meditation

## Abstract

Adherence determines the success and benefits of mental training (e.g., meditation) programs. It is unclear why some participants engage more actively in programs for mental training than others. Understanding neurobiological factors that predict adherence is necessary for understanding elements of learning and to inform better designs for new learning regimens. Clustering patterns in brain networks have been suggested to predict learning performance, but it is unclear whether these patterns contribute to motivational aspects of learning such as adherence. This study tests whether configurations of brain connections in resting-state fMRI scans can be used to predict adherence to two programs: meditation and creative writing. Results indicate that greater system segregation and clustering predict the number of practice sessions and class participation in both programs at a wide range of network thresholds (corrected *p* value < 0.05). At a local level, regions in subcortical circuitry such as striatum and accumbens predicted adherence in all subjects. Furthermore, there were also some important distinctions between groups: Adherence to meditation was predicted by connectivity within local network of the anterior insula and default mode network; and in the writing program, adherence was predicted by network neighborhood of frontal and temporal regions. Four machine learning methods were applied to test the robustness of the brain metric for classifying individual capacity for adherence and yielded reasonable accuracy. Overall, these findings underscore the fact that adherence and the ability to perform prescribed exercises is associated with organizational patterns of brain connectivity.

## Author Summary

Individuals vary in their ability to adhere to learning new skills. We have found that even before individuals undergo training, their brain connectivity patterns are predictive of their capacity to perform the prescribed exercises. We report that variability in brain connectivity contributes to the capacity for adhering to prescribed learning instructions such as meditation. Thus, adherence is associated with the extent of clustering and segregation between known resting-state subnetworks. These findings indicate that system segregation is a determinant feature of the motivation to learn new mental exercises and, more specifically, is conducive for adherence to learning.

## INTRODUCTION

Mental training programs such as mindfulness meditation have become widely accessible for improving cognitive control and emotional regulation (Jha, Stanley, Kiyonaga, Wong, & Gelfand, 2010; Tang et al., 2007; Teper, Segal, & Inzlicht, 2013). Some individuals adhere to the prescribed practice relatively more than others, yet the reasons for this variability remain unclear. The rationale behind mental training programs is that the training methods are accessible and effective for most participants. However, variability in adherence to practive requires further investigation. Notably high and low rates of adherence serve as major factor in curtailing therapy success (Farmer, 1999; Morisky, Green, & Levine, 1986; Osterberg & Blaschke, 2005). This study investigated whether neurobiological factors play a role in mediating individual differences in adherence.

Sustained cognitive focus is an acquired skill that requires considerable effort and commitment. Many people drop out of the practice before reaping any benefits (Bados, Balaguer, & Saldana, 2007). Some participants show more interest and exert maximal effort to follow through on their commitment to practice prescribed tasks, whereas others drop out during the program or perform the tasks only a few times before the study ends. This variability in motivation to perform tasks is a well-known factor in determining the outcomes (Bados et al., 2007; Paas, Tuovinen, van Merrienboer, & Darabi, 2005), but the neurobiological factors for this variability have received little attention. It is hence no surprise that outcomes of mental training exercises vary widely in their effectiveness between individuals (Mathieu, Martineau, & Tannenbaum, 1993) and across studies (Milne, Baker, Blackburn, James, & Reichelt, 1999; Penedo & Dahn, 2005). Factors that may influence attrition and compliance include motivation (Glombiewski, Hartwich-Tersek, & Rief, 2010), discipline (Gong, Rai, Beck, & Heffernan, 2009), innate or previously acquired skill (Dahmann, 2017), or positive expectations (Ryan, Plant, & Omalley, 1995). However, adherence remains difficult to predict and clear mediators of adherence remain largely unknown (Adefolalu, 2018; Holmes, Hughes, & Morrison, 2014; Morisky, Ang, Krousel-Wood, & Ward, 2008).

Cognitive functions are closely linked with the organization of functional connections formed through synchronous fluctuations in neural activity in multiple brain areas (E. Bullmore & Sporns, 2009). Systematic variations in functional connectivity profiles distinguish individuals (Gratton et al., 2018; Kashyap et al., 2019) and have behavioral implications (Hashmi et al., 2014; Just, Cherkassky, Keller, & Minshew, 2004; Kashyap et al., 2019). Functional connections reorganize and segregate into modules differently in individuals, appearing to draw on developmental trajectories (Levitt, 2003; Power, Fair, Schlaggar, & Petersen, 2010), genetic processes (Liu et al., 2009), and prior learning (Maguire, Frith, & Morris, 1999). One such characteristic of brain networks is network segregation, which has been implicated to predict cognitive training success in a few studies (Arnemann et al., 2015; Wig, 2017). High modularity observed at baseline has been shown to predict cognitive training success (Arnemann et al., 2015; Wig, 2017). It was suggested that a preexisting pattern of high segregation in brain connectivity offers a capacity for specialized modules to work independently for allowing better learning performance, thus leading to better training outcomes (Baniqued et al., 2018). Interestingly, the characteristic that predicts cognitive training outcomes also predicts the effects of positively priming expectations towards treatments (Hashmi et al., 2014). The main upshot of these findings is that functional connectivity patterns may be among the determinants of learning outcomes (Gottlich, Kramer, Kordon, Hohagen, & Zurowski, 2015; van Waarde et al., 2015). Moreover, it is not clear whether high clustering and segregation in brain subnetworks facilitates learning per se or whether it is important for motivational aspects during learning. While intrinsic network characteristics may serve a role in facilitating the neurobiological processes required for adhering to prescribed tasks, this link has not yet been tested (Detweiler & Whisman, 1999; Jones, Harris, Waller, & Coggins, 2005).

We hypothesized that intrinsic network properties predict the level of adherence shown by participants to mental training programs. We tested this hypothesis utilizing resting-state fMRI data acquired before healthy participants were pseudorandomized to a meditation program or to a control creative writing program , in a clinical trial which was conducted to test the benefits of meditation. (Greenberg et al., 2018). We investigated global brain properties such as network segregation and mean clustering and also assessed whether the nodes in which clustering potentially predicts adherence are similar or different between the two types of cognitive training programs. Nodes in which high connectivity (degree) predicted adherence were mapped to regions partitioned into five canonical resting state networks. Nodal connectivity patterns found to predict adherence to both of the two programs were taken to represent regions that contribute to adherence irrespective of the differences in the tasks. Nodal connectivity that predicted adherence only to a specific program was taken to be a system that contributes to adherence to the specific task prescribed by that training program. This would likely reflect specific skills related to the task prescribed by each program.

To assess the translational capacity of using such findings to predict adherence, a strategy based on machine learning was implemented. Machine learning is the study of computer algorithms that can learn complex relationships or patterns from data, improve their learning over time in an autonomous manner, and finally make relatively more accurate decisions (Bzdok, Altman, & Krzywinski, 2018; Varoquaux & Thirion, 2014). Thus, we used a few supervised machine learning methods to discriminate high and low adherence to mental training programs by using brain network organizational measures.

## MATERIALS AND METHODS

### Experimental Design

**Figure 1.**
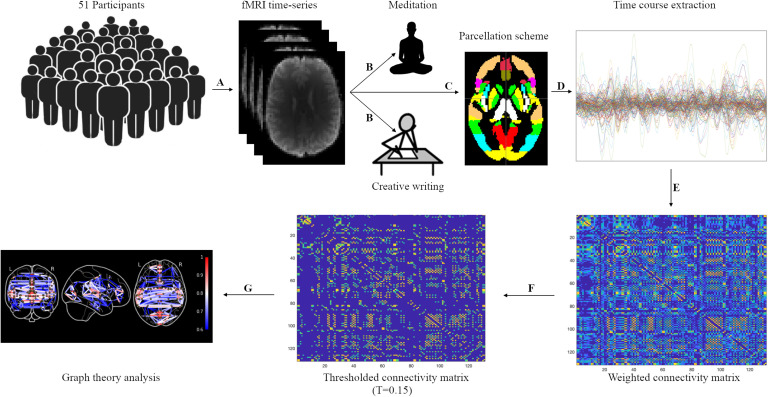
Overview of study design and the pipeline for graph theoretical analysis of resting-state fMRI time series. Healthy participants (*N* = 51, 35 women, age = mean 26) were (A) scanned for structural (T1) and functional MRI resting-state data and (B) randomized to attend a meditation (*n* = 29) or a creative writing (*n* = 22) training program. (C) A set of nonoverlapping brain regions were obtained according to a prior parcellation scheme (optimized Harvard-Oxford) from resting-state functional MRI, (D) averaged time series within regions of interest were extracted, and (E) a weighted interregional correlation matrix was obtained from BOLD time series and (F) was thresholded over a range of thresholds for each participant. (G) The correlation matrix was calculated to assess various graph metrics, and statistical analysis was performed to predict adherence.

This study was originally conducted as a trial to investigate whether mindfulness meditation training improves cognitive performance compared with a creative writing (control) program; a paper with cognitive findings from this study has already been published (Greenberg et al., 2018). Subsequently, participants underwent MRI scans. Participants were asked to attend four weekly web-based classes to receive instructions related to their respective program, and to practice mindfulness or creative writing for 30 min per day 5 times per week. Both the creative writing and the guided meditation home practice materials were provided via a secure web page. This system tracked each participant’s engagement with the practice materials. Adherence criteria in the training program were defined by two measures: (a) the number of classes attended and (b) the number of completed home practice sessions. These data hence proffered an opportunity to measure the connection between adherence and prior brain network states. The schematic of the study design is shown in Figure 1.

### Participants

A total of 51 healthy participants (age = 22–48; 35 females) were scanned using functional magnetic resonance imaging (fMRI). All participants underwent a resting-state scan and were pseudorandomized (by date of baseline testing completion) to a 4-week mindfulness training program (*n* = 29) or a creative writing program (*n* = 22).

Study participants were recruited through fliers and research mailing lists. The inclusion criteria for participants were the following: right handedness, SAT (Scholastic Assessment Test) scores within top 25th percentile (minimal score of 580 verbal, 610 math, 570 writing), and have either completed a 4-year degree or a minimum of 2 years completed in a 4-year degree program in which they were currently enrolled. Additionally, having reliable internet access with a video camera was required, because the training programs and materials were to be administered via the web.

Participants were excluded if they had any neurologic or psychiatric disease, any experience of more than three meditation classes, or participation in more than 20 sessions of mind-body classes such as yoga and tai chi. Psychiatric medication other than a single antidepressant, post-traumatic stress disorder (PTSD) symptomology as assessed by the PTSD checklist-civilian (PCL-C 73), and presence of any MRI contraindicators (e.g., metallic implants, claustrophobia) were other exclusion criteria.

All participants provided their written informed consent as approved by the Institutional Review Board of Massachusetts General Hospital (protocol 2014P000157) and were remunerated up to $150 for completing the study.

### Training Programs

Detailed description of both programs can be found in Greenberg et al. (2018). Briefly, participants in both programs were requested to attend four weekly, web-based training sessions (Cavanagh et al., 2013; Gluck & Maercker, 2011; Harnett, Whittingham, Puhakka, Hodges, & Spry, 2010; Krusche, Cyhlarova, & Williams, 2013), which consisted of 30 min of instruction plus 30 min of practice (meditation or creative writing). All participants were instructed to practice on their own for 30 min per day on 5 non-class days. All home training materials were web-based—guided audio recordings for the meditation class, and writing prompts and response boxes for the creative writing group—so that engagement with these materials could be monitored and recorded electronically.

#### Mindfulness program.

The mindfulness program was led by a trained mindfulness meditation teacher with more than 25 years of mindfulness mediation practice and 4 years’ experience teaching mindfulness meditation. In the first 2 weeks, participants practiced focused-attention meditation, in which attention was focused solely on the breath or body sensations. In the final 2 weeks, they practiced open-monitoring meditation, in which attention was centered on the present experience without predetermining a specific object of focus. The first 15 min of each class were devoted to didactic information about the concept of mindfulness, as well as instruction on how to practice meditation. The last 15 min of each class were devoted to questions and answers from participants about their experiences.

#### Creative writing program.

The creative writing program was structured similarly to the mindfulness program. The program was led by a professional writing tutor with 5 years of tutoring experience. In the first 2 weeks, participants practiced writing a short text about a photo or a short text extracted from Wikipedia.org, in a daily newspaper article format. In the last 2 weeks, they practiced writing in an academic scholarly format. The first 15 min of each class were devoted to didactic information about effective writing techniques, concise written communication, and paragraph structure. The last 15 min of each class were devoted to questions and answers from participants about their experiences.

### MRI Data Acquisition

Whole-brain images were acquired with a 3.0 T Siemens scanner using a 32-channel head matrix coil. T1-weighted images were acquired via standard magnetization-prepared rapid gradient-echo (MPRAGE) sequence in two different dimensions and (TE [echo time] = 1.69 ms, TI = 1,100 ms, FA [flip angle] = 7°; (a) 72 × 72 × 47, 1-mm isotropic voxels, TR [repetition time] = 3 s; (b) 64 × 64 × 37, 1-mm isotropic voxels, TR [repetition time] = 2 s). MPRAGE for high-resolution brain structure (10 min) and a resting-state fMRI (5 min; Mueller et al., 2015) were used in the analysis.

### MRI Data Processing

Preprocessing of resting-state data was performed using in-house BASH scripts that used function libraries from FMRIB Software Library v5.0 (FSL, University of Oxford, United Kingdom; Jenkinson, Beckmann, Behrens, Woolrich, & Smith, 2012), Analysis of Functional Neuroimages (AFNI, National Institute of Mental Health Scientific and Statistical Computing Core, USA; Cox, 1996), and FreeSurfer (Fischl, 2012) software. Steps included correction for head motion, normalization to Montreal Neurological Institute (MNI) space, and smoothing of each fMRI volume with a Gaussian filter.

The following preprocessing steps were applied: The T1-weighted anatomical scans were processed using the “recon-all” tool from FreeSurfer. From recon-all we used the -autorecon1 command to remove unnecessary details of the anatomical image, which could lead to the addition of unneeded features, critical reconstruction, and volumetric segmentation to the next preprocessing step. The -autorecon1 processing stage includes motion correction, registration, nonuniform intensity normalization (NU), Talairach transform computation, intensity normalization 1, and skull strip.

The first five volumes were dropped to allow the blood-oxygen-level-dependent (BOLD) signal to reach a steady state. We used Fourier interpolation to rigid-body motion correction with least squares alignment of each volume to the eighth image. We applied slice-timing correction for interleaved acquisitions between the same slice and voxel in neighboring acquisition TRs. Also, Fourier transformation—which was used to filter temporal bandpass between 0.005 and 0.3 Hz— further filtered out linear and quadratic trends using analysis of functional neuroimages. In addition, FSL was used for spatially smoothing each fMRI volume (Gaussian kernel full width half maximum [FWHM] = 6 mm) and normalizing mean-based intensity.

In the next step, eight nuisance signals consisting of six motion parameters and time courses of white matter and cerebrospinal fluid were regressed out from the data as covariates of no interest. The time series of cerebrospinal fluid and white matter were extracted from masks; this mask obtained from segmentation of individual’s high-resolution structural image thresholded at 80% tissue-type probability. The six motion parameters—for rotational movement around 3 df of a human head (pitch, yaw, and roll axes) and for movement in cardinal directions X, Y, and Z—were generated in an FSL-based motion correction step in native functional space. The registration of functional and structural brain data to the MNI152 template with 2 × 2 × 2 m^3^ resolution was done using FMRIB’s Linear and Nonlinear Image Registration Tools in the following steps: (a) registration of high-resolution structural image to the MNI152 2-mm template with 12 *df* linear affine transformation; (b) registration of functional image to high-resolution structural image with 6 df linear transformation; and (c) registration of functional volume to MNI125 2-mm standard space with structural-to-standard nonlinear transformation matrix. Head motion statistics were calculated by measuring (a) framewise displacement (FD) and (b) motion outlier detection. Participants with FD values greater than 3 mm or motion outliers detected in more than 30% of the acquired data were excluded (Power et al., 2011). None of the participants fit these criteria. In addition, the behavioral findings were correlated with FD values for further verification.

### Brain Parcellation and Network Construction

Brain parcellation was defined as a data clustering problem to group image voxels into clusters. By using a parcellation scheme we can divide the brain’s spatial domain into a set of nonoverlapping regions. Here we used a parcellation scheme that we have previously used (optimized Harvard-Oxford parcellation; 131 regions; Hashmi et al., 2014; Hashmi et al., 2017). To define the brain regions, the preprocessed functional brain images were parcellated into 131 regions of interest (ROIs) using the Harvard-Oxford atlas that divided the brain’s spatial domain into 131 nonoverlapping clusters (see Supplementary Table 1).

These regions are designated as nodes for constructing the graph. The BOLD time series were extracted from each voxel within each node and averaged, resulting in 131 time series points for each participant.

### Computational Modeling of Functional Brain Networks and Graph Construction

A graph analysis approach was used to study segregation and integration in large-scale brain networks (Reijneveld, Ponten, Berendse, & Stam, 2007). The adjacency matrix for each functional dataset was created by pairwise Pearson’s linear correlation coefficient (Gibbons, 1985) from the BOLD time series of each participant to represent 131 × 131 weighted connectivity graph.

Each correlation matrix was thresholded and converted to a binarized adjacency matrix. The process of binarizing a connectivity matrix is based on a predefined correlation threshold (T = 0.05 to 0.5, with steps of 0.05):

$$A_{ij}=\left\{\begin{array}{c} 1,if\;correlation\;matrices\;>\;predefined\;correlation\;threshold\\ 0,\;others \end{array}\right.,$$

where *A*_*ij*_ is a binarized network (van Wijk, Stam, & Daffertshofer, 2010). There is no definitive method for selecting optimal thresholds; hence, it is customary to use a range of possible thresholds over a broad range of values to test for consistency of the results. Threshold values higher than 0.5 carry the risk of overestimation because of noisy, weak, or physiologically insignificant connections. Stringent thresholds, on the other hand, can fragment the networks into a collection of smaller networks that lead to overlooking correlations of functional importance and misrepresenting the graph structure.

We considered brain regions as nodes (*v*_*i*_), and their pairwise connection (edges) represents the relationship between the brain regions (Smith, 2012; Smith et al., 2013). The node is typically considered as brain regions (parcellations of the brain into regions) and edges represent the connection pathway between different regions. Here the graph was described by a binarized connectivity matrix *A*_*ij*_ with entries *a*_*ij*_ = 1 when there is a connection from node *i* to node *j*, and *a*_*ij*_ = 0 if the connection is below the predefined correlation thresholds. A network of brain regions consists of a defined set of nodes, which are linked to each other through edges.

After defining the fMRI correlation matrix, graph theory concepts were used to quantify functional networks of the brain. Thus, the Brain Connectivity Toolbox and custom codes were implemented in MATLAB (Avena-Koenigsberger, Misic, & Sporns, 2018; Rubinov & Sporns, 2010).

#### Brain network analysis.

The relations between nodes and edges in a graph determine the topology of the functional brain networks through a broad array of measures that probe local and global aspects of network organization and the balance between them (Rubinov & Sporns, 2010). The main advantage of using this technique is that it summarizes information from dense and high-dimensional functional connectivity data into single values of network characteristics representing the network. These values capture the extent of segregation and integration that are indicators of balance between cost and efficiency. When mapped to brain regions, graph metrics offer useful information about overall connectivity of nodes and properties of the subnetwork in which they reside. Here we focused primarily on metrics that have been previously demonstrated to predict individual differences in how much pain changed in response to changes in treatment expectations (Hashmi et al., 2014).

These metrics are indicators of the number of modules (modularity), the extent of connectedness within neighbors (clustering coefficient and local efficiency), hub strength (degree centrality), presence of short paths in the global network and how well the graph is connected globally (global efficiency), and system segregation. These metrics are described in detail in the following sections:

*Clustering coefficient*: It is a measure of the amount of clustering in the network. It is the fraction of triangles around a node. The local clustering coefficient in the neighborhood of node *v*_*i*_ is defined as the ratio of actual and maximum possible edges in the graph *G*_*i*_ (Fagiolo, 2007; van den Heuvel & Pol, 2010; Watts & Strogatz, 1998).
  $$C=\frac{1}{n}\sum_{i\in N}C_{i}=\frac{1}{n}\sum_{i\in N}\frac{2L_{i}}{k_{i}\left(k_{i}-1\right)}.$$
  Based on the formula, *C*_*i*_ is the clustering coefficient of node *i*, *K*_*i*_ is the degree of node *i*, *L*_*i*_ is the number of triangles around node *i*, and N is the set of all nodes in the network.
*Global and local efficiencies*: Global efficiency (*E*(*G*)) is a measure of the network’s capacity for parallel information transfer between nodes through multiple series of edges. The average global efficiency of information transfer between the graph *G* having *N* nodes can be calculated from the inverse of the path length *L*_*ij*_ (the edge distances from region *i* to all other regions *j* in the network; Latora & Marchiori, 2001):
  $$E_{glob}=E(G)=\frac{1}{n\left(n-1\right)}\sum_{i\neq j\in G}\frac{1}{L_{ij}}.$$
  Local efficiency for each node *v*_*i*_ is a measure that assesses how efficiently a node can exchange information within its locally connected regions when node *v*_*i*_ is removed. If the subgraph of all neighbors of *v*_*i*_ is denoted by *G*_*i*_, then its local efficiency is approximately equivalent to the clustering coefficient *C*_*i*_ (Achard & Bullmore, 2007).
  $$E_{loc}=\frac{1}{n}\sum_{v_{i}\in G}E\left(G_{i}\right).$$
*Modularity*: Modularity is a measure that describes a set of interconnected subnetworks (modules). A module is a set of densely interconnected nodes that work together and is connected sparsely to the rest of the network (Sporns & Betzel, 2016). Modularity was calculated using the Louvain method for community detection.
  $$Q=\frac{1}{2m}\sum_{i,j}\left[A_{ij}-\frac{k_{i}k_{j}}{2m}\right]d\left(c_{i},c_{j}\right).$$
  The network is partitioned into a set of nonoverlapping modules *m* (total number of modules) defined as following the formula that *A*_*ij*_ represents the weights (0 or 1 in the case of binary network) of all edges between two nodes (*i* and *j*) and *K*_*i*_ = ∑_*j*_*A*_*ij*_ is the sum of the weight of edges attached to node *i*, *c*_*i*_ is the community to which node *i* is belongs, the *δ*-function *δ*(*c*_*i*_, *c*_*j*_) is 1 if *i* = *j* and 0 otherwise *m* = 1/2∑_*ij*_*A*_*ij*_ (Blondel, Guillaume, Lambiotte, & Lefebvre, 2008). The algorithm was tested with 150 repetitions and the resulting mean of Q values was calculated. Since modularity was not related to adherence, network partitions and membership were not explored further.
*Hub analysis and canonical resting-state network analysis*: For hub analysis, we correlated degree (a measure of hubness) with adherence in order to test whether connectivity of particular brain nodes was important for practice.
  Node hubness was measured as degree centrality (*D*_*i*_); degree centrality is defined as the number of connections from the node of interest to other nodes of the network.
  $$D_{i}=\sum_{j\in G}a_{ij},$$
  where *a*_*ij*_ is the *i*th row and *j*th column edge of connectivity matrix *A*_*ij*_. For an individual node, the degree is equal to the number of edges connected to that specific node. The value of degree reflects how important a node is in the brain network. This process is useful in identifying highly connected nodes such as hubs that may play a critical role in information integration. In graph analysis, network hubs are defined as highly connected nodes (nodes with high degree within a network neighborhood; van den Heuvel & Sporns, 2013). Values were computed for different sparsity thresholds, and only those regions that showed a consistent statistical significance after correction for multiple comparisons (false discovery rate, FDR) for at least three thresholds are presented.
  To describe the brain subnetworks involved in predicting adherence to the two different programs, we observed whether the significant nodes for degree and clustering coefficient belonged to specific subnetworks for the meditation or creative writing groups. In addition, we also assessed the network in which the most nodes predicted adherence in all subjects pooled together, that is, irrespective of the type of training. Towards this goal, 131 parcelled brain regions were classified into five known resting-state networks: (a) subcortical, (b) sensory, (c) default mode, (d) attention/executive, and (e) language/memory, using the tool Neurosynth as described previously (Hashmi et al., 2017).
*System segregation for known resting-state networks*: The calculation for system segregation between brain subnetworks was mathematically analogous to the one used previous by Cohen and D’Esposito (2016), except that the connections within subnetworks and between subnetworks are normalized by the total possible number of connections within and between subnetworks using the following formula:
  $$Binarized\;system\;segregation=\frac{{\overset{-}{z}}_{bin_{w}}-{\overset{-}{z}}_{bin_{b}}}{{\overset{-}{z}}_{bin_{w}}}.$$
  Rather than the mean connectivity within a subnetwork, ${\overset{-}{z}}_{{bin}_{w}}$ is the number of connections within a subnetwork, normalized by the total number of possible connections within that subnetwork. This value was then averaged for all subnetworks. Similarly, ${\overset{-}{z}}_{{bin}_{b}}$ is the number of connections between different subnetworks, normalized by the total number of possible connections between subnetworks.

To identify subnetworks, nodes were sorted into five known subnetworks (subcortical, default mode, sensory, attention/executive, and language/memory), as described in the preceding section, to produce affiliation vectors that identified each node’s subnetwork and that were used for the system segregation calculations.

### Statistical Analysis

#### Adherence criteria.

We defined them by the number of total homework assignments completed (total 20 assignments; see the Training Programs section) as well as attendance in online instruction classes (total 4 sessions).

#### Predicting adherence.

First we used permutation tests to establish the correlation between hubness (degree centrality) and clustering coefficients at each node. The *p* values were corrected for multiple comparisons by using FDR set at 0.05 for all threshold values. To find limit analyses to the robustly significant nodes, only nodes that survived after FDR correction at the range of 0.05 to 0.5 in 0.05-increment-tested thresholds were selected.

To compute global metrics, nodal properties (clustering coefficients, local efficiency) were averaged over all nodes, and global metrics such as global efficiency and network segregation were correlated with adherence measures at different sparsity thresholds. The correlation between adherence measures and nodal graph properties are described in the Hub Analysis and Canonical Resting-State Network Analysis section above.

Each of these measures was tested for its relation with adherence (homework assignments completed and attendance) with 1,000 permutations either in all participants, or separately for the mindfulness group or creative writing group. The number of independent tests was corrected for all graph measures and all tests were thresholded using FDR for each condition and as reported before.

### Machine Learning–Based Prediction of Adherence

Machine learning was used to predict adherence to the mental training programs. Data-driven prediction may indicate the most probable behavior expected from a person. The basic assumption used is that fMRI signals taken from a human’s brain exhibit a spatial pattern that contains information about an individual’s behavioral states.

Classifying adherence with brain network organization measures: We used the nodal measures since these measures were most strongly predictive of adherence (clustering coefficient and degree centrality). Among the 131 nodes, we used only the significant nodes listed in Tables 3 and 4. Our criteria were guided by previous recommendations as our features for the machine learning model (Bzdok, Krzywinski, & Altman, 2017).

For this approach, we applied four different classifiers: random forest, AdaBoost (Adaptive Boosting), decision tree, and Naïve Bayes. Random forest and AdaBoost are among frequently used classifiers that discriminate between classes using ensembles of atomic classifiers (J. C. W. Chan & Paelinckx, 2008). A random forest classifier (Breiman, 2001) includes several decision trees randomly built from provided features; this classifier works based on a consensus between its trees. AdaBoost classifier (Freund & Schapire, 1996) focuses on classification problems and attempts to convert a set of weak classifiers into a strong one. A decision tree is a graph that classifies the data by using a tree-like model to illustrate every possible outcome of a decision (Quinlan, 1986). The Naïve Bayes classifier (Rish, 2001) assumes that the effect of a feature on given class is independent (naïve assumption) of the values of other features. Considering classification under a low-data regime, a lower number of features are desirable to learn a simpler model that does not overfit to the scarce data. We selected the most important features for each classifier using the greedy method.

We used backward elimination to prune features as described in Horst and Macewan (1960). It starts evaluation using all features and eliminates the ones that are less impactful on the performance of a random forest classifier consisting of 100 trees.

The classification is done in a binary setup, with individuals who completed their homework more than 10 times falling into one class (high) and the rest considered as the other class (low). A quarter of participants were randomly selected and separated as the hold-out set in a stratified order. Using the rest of the data, classifiers are trained using leave-one-out cross-validation (LOOCV) repeated with five different seeds. The 20 scores acquired for each classifier were averaged and reported with 95% confidence. Finally, the trained classifiers were tested on the held-out set.

Since data acquisition in human neuroimaging studies is costly, the number of total participants is generally limited. Also, by removing a part of data for the held-out set, the number of training data will be reduced and the risk of underfitting will be increased. Thus, generalization ability that is afflicted by the small number of train and test sample sizes can be improved by verifying the classifier’s accuracy with a cross-validation procedure (Kohavi, 1995). One of the frequently used methods in such a situation is k-fold cross-validation (k-fold CV; Bengio & Grandvalet, 2004).

In k-fold CV, the training sample data are divided into k-folds as equally as possible. Since the sample size is small, we used leave-one-out cross-validation (Patel, Khalaf, & Aizenstein, 2016). The LOOCV strategy is the spatial case of k-fold CV, where *k* is equal to the training sample size for estimating the prediction performance (Hastie, Tibshirani, & Friedman, 2013). In each cross-validation trial, one sample was left out for the test, the remaining samples were used for fitting the classifier, and the fitted classifier was employed to predict the left-out sample. This process was repeated for all training sample size.

The receiver operating characteristic (ROC) method was used to assess the performance and efficacy of classifiers on classification model (Fernandez-Lozano et al., 2015). ROC curves were generated based on the true positive (TP, sensitivity) rate versus false positive (FP) rate (Ragab, Noaman, Al-Ghamdi, & Madbouly, 2014). For estimating the reliability of each method, we used the area under the ROC curve (AUC) as a score. The AUC score is between 0 and 1, and a perfect classifier will achieve an AUC of 1 (Stewart, Nuthmann, & Sanguinetti, 2014).

## RESULTS

### Demographic Distribution and Level of Adherence

Descriptive information showing the demographic and baseline characteristic of participants in all participants pooled together, and the creative writing and mindfulness as subgroups separately are shown in Table 1.

**Table 1.** Demographic and baseline characteristics of participants. A total of 51 participants were randomly separated into two types of mental training programs: a meditation group (*n* = 29) or a creative writing program (*n* = 22).

**Parameters**	**All participants** (*N* = 51)	**Creative writing** (*n* = 22)	**Meditation** (*n* = 29)
Age (years)	18–48	18–48	19–48
Total homework	1–20	1–18	1–20
Attendance	1–4	1–4	1–4
Sex	F = 35, M = 16	F = 16, M = 6	F = 19, M = 10
Years of education	12–22	13–20.5	12–22

On assessing the variability in adherence, we found that homework (mean = 9.92 assignments, *SD* = 5.796) and class attendance (mean = 2.92 sessions, *SD* = 0.91) varied significantly between participants and were normally distributed. The number of homework assignments completed ranged from a minimum of 1 to a maximum of 20 out of a total number of 4 classes. There was no significant difference in classes attended or homework assignment between participants randomized to meditation or creative writing groups (corrected *p* value ≤ 0.05, *t* tests).

To examine whether demographic factors (sex and age) have influence on adherence criteria, we used multivariate analysis of variance (MANOVA). The MANOVA was conducted with age and sex (male and female) as factors and using adherence criteria (attendance and total homework) as dependent variables. There was no statistically significant difference for demographic factors. Also, post hoc comparisons of demographic factors were insignificant for attendance and total homework.

### Role of Regional Network Organization in Predicting Adherence

**Figure 2.**
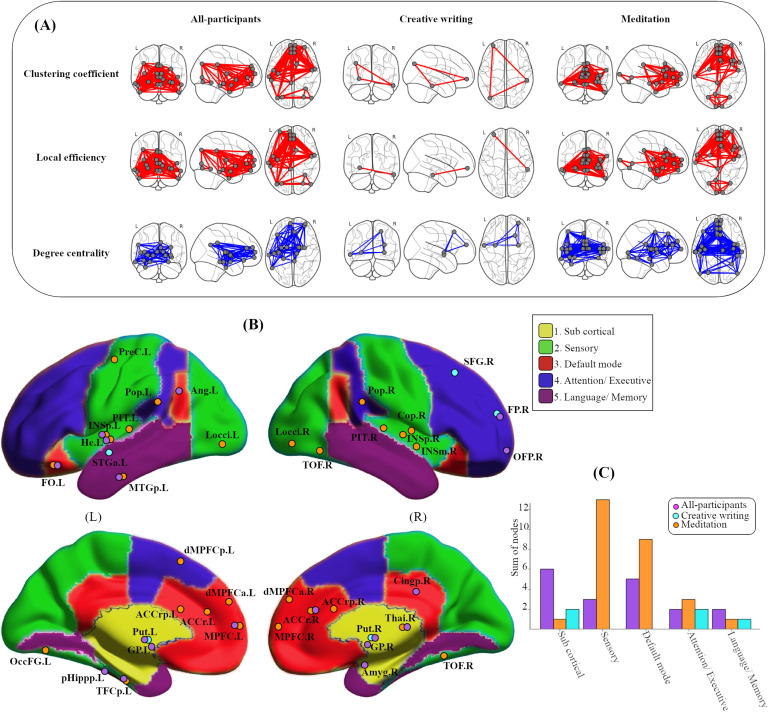
Predicting adherence based on regional connectivity. (A) Spatial pattern of the brain connection for all participants pooled together, creative writing and meditation groups plotted during resting-state fMRI acquisition based on relationship between graph properties and homework. Glass brain images are showing regions (circles) in which graph metrics significantly predicted adherence. The red lines represent edges (connections) between the significant nodes at threshold (T = 0.35). The top red-colored glass brains represent clustering connections; the middle ones are based on local efficiency; and the blue-colored glass brains are based on spatial distribution and connectivity pattern of homework adherence and hubness as degree centrality. (B) The complete parcellation scheme consisted of 131 regions that mapped to five resting-state networks listed in the legend. The circles represent the regions of the brain that were predictive for each group. Overlapped circles indicate that the region was significant for both groups. (C) The bar graph represents the sum of nodes available for all participants, creative writing, and meditation groups based on the five known resting-state brain networks.

First, to test whether optimizations in connectivity (clustering coefficient and local efficiency) observed in intrinsic networks predict individual variability in adherence, nodewise metrics were correlated with the total number of home practice assignments using a permutation test corrected for multiple comparisons. Both local efficiency and clustering were significant predictors of practice showing similar results. There was significant spatial overlap between these metrics, with a few variations (See Figure 2A/red and Table 2).

**Table 2.** Brain regions that predict adherence to practice (total homework) based on clustering coefficients, local efficiency, and degree centrality. (Statistical significance is based on permutation test calculated at corrected *p* value = 0.05 and also corrected for multiple comparisons.) Shown are regions based on the optimized Harvard-Oxford atlas, along with abbreviations and x, y, z MNI coordinates based on center of mass followed by Pearson R values and permuted corrected *p* values.

**Regions**	**Abbreviations**	**x**	**y**	**z**	**r**	**p**
**Clustering coefficient: All participants**						
Caudal anterior cingulate left	ACCc_L	−4	40	−2	0.3223	0.031
Caudal anterior cingulate right	ACCc_R	4	40	−2	0.2778	0.046
Cingulate gyrus, posterior division left	Cingp_L	−4	−38	32	0.3101	0.024
Dorsal medial prefrontal cortex, anterior division left	dMPFCa_L	−4	50	28	0.3319	0.018
Dorsal medial prefrontal cortex, anterior division right	dMPFCa_R	4	50	28	0.3168	0.023
Frontal pole right	FP_R	30	54	20	0.3124	0.022
Posterior insula left	INSp_L	−38	−14	8	0.3036	0.027
Inferior temporal gyrus, anterior division left	ITGa_L	−50	−6	−40	0.3176	0.023
Inferior temporal gyrus, anterior division right	ITGa_R	50	−6	−40	0.3129	0.023
Inferior temporal gyrus, posterior division left	ITGp_L	−56	−32	−24	0.3589	0.009
Lateral occipital cortex, inferior division right	LOcci_R	48	−78	−2	0.3632	0.015
Lateral occipital cortex, superior division left	LOccs_L	−40	−78	34	0.3033	0.031
Medial prefrontal cortex left	MPFC_L	−6	60	8	0.2925	0.038
Medial prefrontal cortex right	MPFC_R	6	60	8	0.309	0.025
Middle temporal gyrus, anterior division right	MTGa_R	58	−2	−22	0.3746	0.007
Middle temporal gyrus, posterior division left	MTGp_L	−62	−22	−18	0.3676	0.008
Nucleus accumbens right	NAc_R	10	10	−8	−0.4049	0.003
Occipital fusiform gyrus left	OccFG_L	−28	−76	−14	0.434	0.002
Occipital fusiform gyrus right	OccFG_R	28	−76	−14	0.3637	0.01
Temporal occipital fusiform cortex right	TOF_R	34	−54	−16	0.4508	0.002
Ventral medial prefrontal cortex left	vMPFC_L	−4	50	−20	0.2979	0.04
Ventral medial prefrontal cortex right	vMPFC_R	4	50	−20	0.3649	0.01
**Meditation**						
Caudal anterior cingulate left	ACCc_L	−4	40	−2	0.6899	9.99E-04
Caudal anterior cingulate right	ACCc_R	4	40	−2	0.683	0.002
Rostral anterior cingulate left	ACCr_L	−4	38	18	0.4839	0.013
Rostral anterior cingulate mid posterior right	ACCcrm_R	6	18	34	0.3898	0.048
Rostral anterior cingulate posterior right	ACCrp_R	4	22	20	0.3747	0.047
Rostral anterior cingulate right	ACCr_R	4	38	18	0.4755	0.016
Amygdala left	Amyg_L	−24	−4	−18	−0.4271	0.021
Cingulate gyrus, posterior division left	Cingp_L	−4	−38	32	0.4557	0.017
Dorsal anterior insula right	dINsa_R	32	20	0	0.4851	0.007
Frontal operculum cortex right	Fop_R	40	20	4	0.5316	0.003
Inferior frontal gyrus, pars opercularis right	IFGpo_R	54	14	16	0.4221	0.016
Inferior temporal gyrus, anterior division right	ITGa_R	50	−6	−40	0.4725	0.016
Lingual gyrus left	Ling_L	−10	−68	−2	0.4461	0.019
Medial prefrontal cortex left	MPFC_L	−6	60	8	0.3772	0.049
Medial prefrontal cortex right	MPFC_R	6	60	8	0.4389	0.025
Middle temporal gyrus, posterior division left	MTGp_L	−62	−22	−18	0.4236	0.037
Occipital fusiform gyrus left	OccFG_L	−28	−76	−14	0.4572	0.009
Occipital fusiform gyrus right	OccFG_R	28	−76	−14	0.4859	0.011
Occipital pole left	OccP_L	−8	−100	6	0.58	9.99E-04
Occipital pole right	OccP_R	8	−100	6	0.5708	0.003
Orbito frontal pole right	OFP_R	32	58	−6	0.4461	0.015
Ventral anterior insula right	vINsa_R	36	10	−14	0.451	0.011
Ventral medial prefrontal cortex left	vMPFC_L	−4	50	−20	0.5291	0.004
Ventral medial prefrontal cortex right	vMPFC_R	4	50	−20	0.4445	0.018
**Creative writing**						
Inferior temporal gyrus, posterior division right	ITGp_R	56	−32	−24	0.4892	0.022
Lateral occipital cortex, superior division left	LOccs_L	−40	−78	34	0.4915	0.021
Orbito frontal pole left	OFP_L	−32	58	−6	0.5723	0.009
**Local efficiency: All participants**						
Caudal anterior cingulate left	ACCc_L	−4	40	−2	0.3629	0.01
Caudal anterior cingulate right	ACCc_R	4	40	−2	0.3366	0.014
Cingulate gyrus, posterior division left	Cingp_L	−4	−38	32	0.3025	0.036
Dorsal medial prefrontal cortex, anterior division left	dMPFCa_L	−4	50	28	0.4052	0.005
Dorsal medial prefrontal cortex, anterior division right	dMPFCa_R	4	50	28	0.3126	0.036
Posterior insula left	INSp_L	−38	−14	8	0.3239	0.016
Inferior temporal gyrus, anterior division left	ITGa_L	−50	−6	−40	0.3396	0.019
Inferior temporal gyrus, anterior division right	ITGa_R	50	−6	−40	0.3065	0.037
Inferior temporal gyrus, posterior division left	ITGp_L	−56	−32	−24	0.3124	0.027
Lateral occipital cortex, inferior division right	LOcci_R	48	−78	−2	0.3312	0.017
Lateral occipital cortex, superior division left	LOccs_L	−40	−78	34	0.306	0.032
Medial prefrontal cortex left	MPFC_L	−6	60	8	0.3407	0.017
Middle temporal gyrus, anterior division right	MTGa_R	58	−2	−22	0.3092	0.031
Middle temporal gyrus, posterior division left	MTGp_L	−62	−22	−18	0.4472	9.99E-04
Nucleus accumbens right	NAc_R	10	10	−8	−0.3448	0.02
Occipital fusiform gyrus left	OccFG_L	−28	−76	−14	0.3833	0.002
Occipital fusiform gyrus right	OccFG_R	28	−76	−14	0.3642	0.015
Orbito frontal pole left	OFP_L	−32	58	−6	0.2855	0.038
Temporal occipital fusiform cortex right	TOF_R	34	−54	−16	0.4256	9.99E-04
Ventral medial prefrontal cortex left	vMPFC_L	−4	50	−20	0.303	0.035
Ventral medial prefrontal cortex right	vMPFC_R	4	50	−20	0.2905	0.037
**Meditation**						
Caudal anterior cingulate left	ACCc_L	−4	40	−2	0.6899	9.99E-04
Caudal anterior cingulate right	ACCc_R	4	40	−2	0.683	9.99E-04
Rostral anterior cingulate left	ACCr_L	−4	38	18	0.4839	0.007
Rostral anterior cingulate mid posterior right	ACCcrm_R	6	18	34	0.4219	0.021
Rostral anterior cingulate posterior right	ACCrp_R	4	22	20	0.4226	0.016
Rostral anterior cingulate right	ACCr_R	4	38	18	0.4755	0.008
Amygdala left	Amyg_L	−24	−4	−18	−0.4458	0.022
Cingulate gyrus, posterior division left	Cingp_L	−4	−38	32	0.4481	0.014
Frontal operculum cortex right	Fop_R	40	20	4	0.5387	0.002
Inferior frontal gyrus, pars opercularis right	IFGpo_R	54	14	16	0.394	0.044
Posterior insula right	INSp_R	38	−14	8	−0.3792	0.044
Inferior temporal gyrus, anterior division right	ITGa_R	50	−6	−40	0.4701	0.011
Lingual gyrus left	Ling_L	−10	−68	−2	0.4728	0.011
Medial prefrontal cortex left	MPFC_L	−6	60	8	0.4352	0.014
Medial prefrontal cortex right	MPFC_R	6	60	8	0.5325	0.003
Middle temporal gyrus, anterior division left	MTGa_L	−58	−2	−22	0.475	0.008
Middle temporal gyrus, posterior division left	MTGp_L	−62	−22	−18	0.5004	0.006
Occipital fusiform gyrus left	OccFG_L	−28	−76	−14	0.4528	0.014
Occipital fusiform gyrus right	OccFG_R	28	−76	−14	0.409	0.026
Occipital pole left	OccP_L	−8	−100	6	0.6165	0.002
Occipital pole right	OccP_R	8	−100	6	0.5689	0.002
Orbito frontal pole right	OFP_R	32	58	−6	0.4424	0.016
Ventral anterior insula right	vINsa_R	36	10	−14	0.4518	0.016
Ventral medial prefrontal cortex left	vMPFC_L	−4	50	−20	0.4663	0.013
Ventral medial prefrontal cortex right	vMPFC_R	4	50	−20	0.4817	0.014
**Creative writing**						
Inferior temporal gyrus, posterior division right	ITGp_R	56	−32	−24	0.5137	0.009
Orbito frontal pole left	OFP_L	−32	58	−6	0.4847	0.025
**Degree centrality: All participants**						
Rostral anterior cingulate right	ACCr_R	4	38	18	0.2918	0.038
Amygdala right	Amyg_R	24	−4	−18	−0.3268	0.019
Angular gyrus left	Ang_L	−54	−56	26	0.277	0.045
Cingulate gyrus, posterior division right	Cingp_R	4	−38	32	−0.3111	0.028
Frontal orbital cortex left	FO_L	−40	30	−14	0.3108	0.033
Frontal pole right	FP_R	30	54	20	0.2874	0.043
Globus pallidus left	GP_L	−16	−2	−2	−0.2883	0.037
Globus pallidus right	GP_R	16	−2	−2	−0.3767	0.008
Heschls gyrus (includes H1 and H2) left	He_L	−48	−18	6	−0.2632	0.05
Posterior insula left	INSp_L	−38	−14	8	−0.3042	0.04
Medial prefrontal cortex left	MPFC_L	−6	60	8	0.2975	0.029
Middle temporal gyrus, posterior division left	MTGp_L	−62	−22	−18	0.3302	0.019
Orbito frontal pole right	OFP_R	32	58	−6	0.2862	0.046
Parahippocampal gyrus, posterior division left	pHippp_L	−24	−32	−18	−0.3074	0.034
Putamen left	Put_L	−30	−4	0	−0.3424	0.015
Putamen right	Put_R	30	−4	0	−0.3353	0.022
Temporal fusiform cortex, posterior division left	TFCp_L	−36	−16	−32	−0.3022	0.038
Thalamus right	Thal_R	10	−18	8	−0.3524	0.014
**Meditation**						
Rostral anterior cingulate left	ACCr_L	−4	38	18	0.6394	0.001
Rostral anterior cingulate posterior left	ACCrp_L	−4	22	20	0.5768	0.002
Rostral anterior cingulate posterior right	ACCrp_R	4	22	20	0.455	0.011
Rostral anterior cingulate right	ACCr_R	4	38	18	0.6948	0.001
Central opercular cortex right	Cop_R	48	−4	8	−0.4922	0.01
Dorsal medial prefrontal cortex, anterior division left	dMPFCa_L	−4	50	28	0.4904	0.008
Dorsal medial prefrontal cortex, anterior division right	dMPFCa_R	4	50	28	0.5203	0.005
Dorsal medial prefrontal cortex, posterior division left	dMPFCp_L	−4	26	48	0.3831	0.042
Frontal orbital cortex left	FO_L	−40	30	−14	0.4285	0.019
Heschls gyrus (includes H1 and H2) left	He_L	−48	−18	6	−0.4489	0.02
Middle insula right	INSm_R	40	−2	−2	−0.5326	0.006
Posterior insula left	INSp_L	−38	−14	8	−0.3825	0.03
Posterior insula right	INSp_R	38	−14	8	−0.3784	0.041
Lateral occipital cortex, inferior division left	LOcci_L	−48	−78	−2	−0.4794	0.008
Lateral occipital cortex, inferior division right	LOcci_R	48	−78	−2	−0.372	0.042
Medial prefrontal cortex left	MPFC_L	−6	60	8	0.5845	0.002
Medial prefrontal cortex right	MPFC_R	6	60	8	0.5837	0.003
Middle temporal gyrus, posterior division left	MTGp_L	−62	−22	−18	0.3702	0.048
Occipital fusiform gyrus left	OccFG_L	−28	−76	−14	−0.386	0.039
Planum temporale left	PlT_L	−60	−22	8	−0.4071	0.018
Planum temporale right	PlT_R	60	−22	8	−0.377	0.03
Parietal operculum cortex left	Pop_L	−48	−32	20	−0.5165	0.005
Parietal operculum cortex right	Pop_R	48	−32	20	−0.4545	0.014
Precentral gyrus left	PreC_L	−44	−8	52	−0.3989	0.037
Temporal fusiform cortex, posterior division left	TFCp_L	−36	−16	−32	−0.4772	0.008
Thalamus right	Thal_R	10	−18	8	−0.4081	0.034
Temporal occipital fusiform cortex right	TOF_R	34	−54	−16	−0.4639	0.009
**Creative writing**						
Frontal pole right	FP_R	30	54	20	0.4576	0.033
Putamen left	Put_L	−30	−4	0	−0.4631	0.03
Putamen right	Put_R	30	−4	0	−0.4543	0.027
Superior frontal gyrus right	SFG_R	22	22	54	0.4365	0.048
Superior temporal gyrus, anterior division left	STGa_L	−58	−4	−6	−0.4434	0.039

Second, to identify the nodes that contributed to predicting adherence and determining whether they were specific to the type of practice, we identified the nodes that showed a significant relation between connectivity (degree) and adherence (total hours of practice) and subsequently mapped their affiliations to known resting-state networks. The connectivity of several nodes (degree) was predictive of the number of times participants practiced at home (Figure 2A/blue). However, there were clear differences in nodal affiliation in the three groups (Figure 2C). As shown in Figure 2B, nodes that predicted adherence to meditation practice were predominantly affiliated with brain regions within the default mode network, monitoring and sensory regions such as the anterior cingulate and anterior insula. In contrast, only a few regions significantly predicted adherence to creative writing. The creative writing practice was predicted by only a few nodes in subcortical, attention, and language/memory networks. For all participants combined, there was a clear affiliation of predictive nodes with regions in subcortical areas. The significant nodes overlapped between the groups in a few regions, which for the most part belonged to different regions. For detailed names and strength of correlations (R and corrected *p* values), see Table 2.

### Predicting Adherence With Globally Integrated Graph Measures

At this stage, instead of nodewise metrics, we used globally integrated measures of network integration (mean clustering coefficients, mean local efficiency, and modularity) and segregation (global efficiency) to investigate whether these measures can be used to predict adherence (based on the total number of home practice assignments measured at corrected *p* value ≤ 0.05). The relationship of these integrated values was consistent across domains.

**Figure 3.**
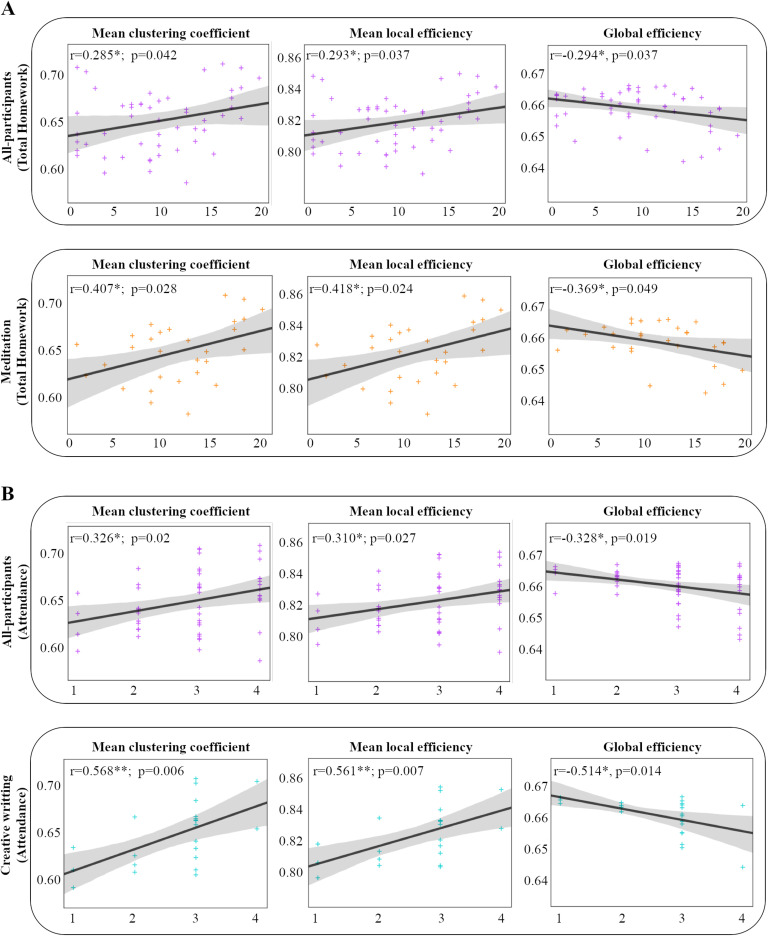
Scatterplots of the relationships between graph metrics and adherence criteria (for the correlation observed at threshold, T = 0.35) corrected at *p* value ≤ 0.05. (A) Based on total homework, for results pooled from all participants together and separately for the meditation group. (B) Based on attendance, for results pooled from all participants and separately for creative writing group. Higher clustering and local efficiency and less integration (global efficiency) and high system segregation (not shown; see Table 3) in brain connectivity in resting-state fMRI predicted adherence. The shaded area shows confidence interval.

Higher clustering coefficients and local efficiency predicted higher levels of adherence (Figure 3A, Table 3) when results were pooled for the two groups when measured at a range of network sparsity thresholds (T = 0.05 to 0.5, corrected *p* value ≤ 0.05); the results were significant after correction of multiple comparisons. In contrast, a lower global efficiency and high system segregation was a significant predictor of more hours spent practicing. Permutation tests using optimized Harvard-Oxford parcellation in networks held at 10 different thresholds (T = 0.05 to 0.5) corrected at *p* value ≤ 0.05 showed consistent results. The results for the subgroup that practiced meditation were similar to those of all subjects pooled together. However, the number of practice sessions by the creative writing group was predicted primarily by the measure of integration (global efficiency) and was less robust relative to the meditation groups or the all-participants group.

**Table 3.** Prediction of adherence based on graph properties averaged from the entire network. The Pearson correlation with 1,000-permutation test was calculated in 10 thresholds (T = 0.05 to 0.5 with steps of 0.05); correlation between the segregation and integration measures of graph theory and the total number of completed homework assignments measured at corrected *p* value ≤ 0.05. Only the p values that remained significant after correction for multiple comparisons at a false discovery rate (FDR) of 0.05 were considered.

Group	Threshold	R value	Corrected *p* value	Q value
All participants	Mean clustering coefficient			
	0.35	0.2853	0.039	0.006
	0.4	0.2919	0.039	0.006
	0.45	0.2983	0.033	0.006
	0.5	0.299	0.039	0.006
	Global efficiency			
	0.35	−0.2936	0.037	0.006
	0.4	−0.3268	0.015	0.006
	0.45	−0.3717	0.005	0.006
	0.5	−0.321	0.02	0.006
	Mean local efficiency			
	0.3	0.2884	0.038	0.006
	0.35	0.2929	0.034	0.006
	0.4	0.2973	0.033	0.006
	0.45	0.2985	0.035	0.006
	0.5	0.2981	0.038	0.006
	System segregation			
	0.1	0.331	0.018	0.006
	0.15	0.330	0.019	0.006
	0.2	0.360	0.010	0.006
	0.25	0.373	0.007	0.006
	0.3	0.394	0.004	0.006
	0.35	0.391	0.005	0.006
	0.4	0.386	0.005	0.006
	0.45	0.380	0.006	0.006
	0.5	0.370	0.007	0.006
Meditation	Mean clustering coefficient			
	0.25	0.3827	0.048	0.0308
	0.3	0.4351	0.018	0.0308
	0.35	0.4074	0.03	0.0308
	0.4	0.387	0.042	0.0308
	Global efficiency			
	0.3	−0.3736	0.039	0.0308
	0.35	−0.3986	0.043	0.0308
	0.4	−0.3809	0.031	0.0308
	0.45	−0.3497	0.046	0.0308
	Mean local efficiency			
	0.25	0.3958	0.034	0.0308
	0.3	0.4675	0.014	0.0308
	0.35	0.4181	0.027	0.0308
	0.4	0.3909	0.043	0.0308
	System segregation			
	0.2	0.381	0.042	0.0308
	0.25	0.402	0.031	0.0308
	0.3	0.415	0.025	0.0308
	0.35	0.413	0.026	0.0308
	0.4	0.415	0.025	0.0308
	0.45	0.412	0.026	0.0308
	0.5	0.409	0.028	0.0308
Creative writing	Global efficiency			
	0.45	−0.4577	0.023	0.0137
	0.5	−0.4966	0.019	0.0137

### Predicting Attendance of Instructional Classes as a Second Domain of Adherence

As a further confirmation of the link between brain network organization and adherence, the network measures used for predicting hours spent at practice were also used to predict attendance at instructional classes.

Attendance compliance showed a similar pattern as homework practice compliance for all participants pooled together and was predicted positively by the measures of segregation and negatively by the measure of integration. Results from the creative writing group were significant at a range of network sparsity thresholds (0.05–0.5, corrected *p* value ≤ 0.05), but the subgroup that practiced meditation did not show any significant results (Figure 3B, Table 4). Significant correlations with framewise displacement could not be detected for any of the brain measures or behavioral variables predicted by the brain measures (*p* < 0.05, uncorrected).

**Table 4.** Calculating the correlation between the segregation and integration measures of graph theory and class attendance based on Pearson correlation with 1,000-permutation test in 10 thresholds (T = 0.05 to 0.5 with steps of 0.05) corrected at *p* value ≤ 0.05. The results that remained significant after correction for multiple comparisons at a false discovery rate (FDR) of 0.05 were considered.

Group	Threshold	R value	Corrected *p* value	Q value
All participants	Mean clustering coefficient			
	0.25	0.2938	0.041	0.0076
	0.3	0.315	0.027	0.0055
	0.35	0.3261	0.023	0.0052
	0.4	0.3208	0.022	0.0052
	0.45	0.322	0.02	0.0052
	0.5	0.3226	0.018	0.0052
	Global efficiency			
	0.1	−0.286	0.049	0.0078
	0.15	−0.3264	0.022	0.0052
	0.2	−0.3576	0.012	0.0052
	0.25	−0.3562	0.011	0.0052
	0.3	−0.3273	0.021	0.0052
	0.35	−0.3282	0.022	0.0052
	0.4	−0.331	0.021	0.0052
	0.45	−0.341	0.02	0.0052
	0.5	−0.2815	0.046	0.0077
	Mean local efficiency			
	0.3	0.2856	0.043	0.0076
	0.35	0.3104	0.028	0.0055
	0.4	0.317	0.021	0.0052
	0.45	0.3192	0.017	0.0052
	0.5	0.321	0.017	0.0052
Creative writing	Mean clustering coefficient			
	0.25	0.4972	0.024	0.0106
	0.3	0.5525	0.009	0.006
	0.35	0.5681	0.009	0.006
	0.4	0.5561	0.009	0.006
	0.45	0.5557	0.009	0.006
	0.5	0.5424	0.012	0.0067
	Global efficiency			
	0.15	−0.4714	0.03	0.0121
	0.2	−0.5771	0.003	0.006
	0.25	−0.6175	0.001	0.006
	0.3	−0.5408	0.013	0.0067
	0.35	−0.5143	0.02	0.0095
	0.4	−0.4758	0.031	0.0121
	0.45	−0.4466	0.039	0.0137
	Mean local efficiency			
	0.25	0.4622	0.037	0.0137
	0.3	0.5352	0.01	0.006
	0.35	0.5605	0.008	0.006
	0.4	0.5514	0.009	0.006
	0.45	0.554	0.01	0.006
	0.5	0.542	0.01	0.006

### Predicting Adherence to Behavioral Practice Using Machine Learning

We compared different classifiers (random forest, AdaBoost, decision tree, and Naïve Bayes) to classify and predict participants’ ability to adhere to the behavioral training practice. For this purpose, we separated individuals into two groups based on the number of practice hours (less than 10 assignments defined as low homework). Determining the level of adherence to behavioral training courses was defined as a binary (high/low homework) classification problem. Backward elimination method was used to reduce the number of preexisting features and to reduce the dimensionality of the model.

**Figure 4.**
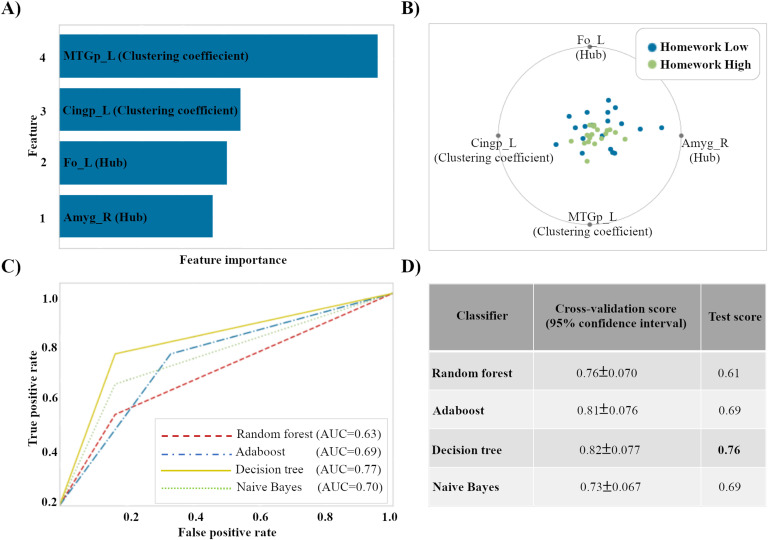
Predicting adherence based on machine learning perspective. (A) Effective features based on the feature selection method. (B) Regional connectivity measures of the brain selected by the backward elimination method for predicting adherence. Blue and green points on this figure represent low and high classes, respectively. (C) ROC curve for four different classifiers for predicting adherence to mental training programs. As we can see, decision tree shows higher area under curve (AUC = 0.77) compared with other classifiers. (D) Comparison of the score of different classifiers on predicting adherence to behavioral training course (prediction based on nodal measures of resting-state fMRI).

Figure 4B shows visual discrimination between classes (high/low homework) based on the features that have high influence on adherence in mental training programs. The generalization of the model to unseen data was tested with a held-out set containing 25% of the whole dataset (no overlap with training set). Also, the performance of the classifiers was evaluated using LOOCV. Based on Figure 4D, decision tree was found to generalize better than the other three models and is less likely to overfit for adherence classification where it uses features selected from regional connectivity of resting-state fMRI scans (cross-validation score = 0.82±0.077%, test score = 0.76%).

We compute the ROC curves of the machine learning algorithm to visualize the classifiers’ performance. In Figure 4C we show the results of the ROC curve. The curves show the predictive ability for classifiers. The classifier had larger AUC, which implied higher sensitivity and specificity. We used the area under the ROC curve, AUC, as a performance measure for machine learning algorithms. We evaluated four machine algorithms (random forest, AdaBoost, decision tree, and Naïve Bayes) on our hypothesis. The AUC values for all four classifiers are given as follows: random forest = 0.63, AdaBoost = 0.69, decision tree = 0.77, and Naïve Bayes = 0.70. As observed from the figure, the decision tree classifier (with AUC = 0.77) showed higher sensitivity and specificity.

## DISCUSSION

We investigated whether brain network configurations can predict a person’s ability to adhere to mental training programs. We report that higher segregation and clustering in resting-state brain networks measured before training can predict adherence to training. The results were reproducible and consistent when tested across variables, and the predictive measures were demonstrably useful for classification with a machine learning approach. The findings indicate a functional significance of brain network organization as a factor for participants’ adherence and engagement in learning new mental skills such as meditation. Support for our investigation comes from studies by Mascaro et al. (2018) and Mascaro, Rilling, Negi, and Raison (2013), which show that preexisting brain activation has the capacity to predict future engagement such as subsequent practice time and class attendance.

Network properties are useful for summarizing and understanding significant functional connectivity patterns of the brain. Higher optimizations within local networks (clusters) and the presence of fewer short paths between clusters are indicative of greater independence of the local network. These properties have already received significant traction in studies on cognitive functions such as working memory, intelligence, and learning (Arnemann et al., 2015; Cohen & D’Esposito, 2016; Stevens, Tappon, Garg, & Fair, 2012). In recent years, converging evidence has linked network segregation to learning success, but the exact mental or mechanistic processes supported by network segregation remain unclear. Several neuroimaging studies have now shown that individual differences in the organization of structural and functional links in the brain can be predictive of individual differences in performance on different types of training programs designed for improving cognition (Tompson, Falk, Vettel, & Bassett, 2018). Cognitive gains in executive function after a physical exercise program were predicted by high modularity in baseline resting state (Baniqued et al., 2018). Improvements in cognition after a cognitive training program could be predicted based on segregation and modularity in intrinsic networks observed at baseline (Gallen et al., 2016). Another significant connection was highlighted in a study by Mattar et al. (2018), in which high independence between two modules, defined for its role in motor learning observed at baseline, was predictive of better learning performance on a motor task. Taken together with the present findings, it appears that network segregation and clustering are among the determinants of adherence to learning.

Another notable link between network clustering and learning was observed where high clustering predicts learning outcomes not in cognition but in top-down pain relief induced by expectations: we feel less pain when we expect less pain (Hashmi et al., 2014). Expectations result from predictions generated from prior conditioning and associative learning. Effects of this type of learning could be predicted with high clustering in baseline resting-state fMRI data. The effect was mediated by brain nodes responsible for learning, motivation, emotional appraisal, and top-down cognition. When observed in lieu of previous findings, the present evidence directs us to a role of organizational patterns of connections of brain clusters in some aspect related to motivation to learn (Fagiolo, 2007; van den Heuvel & Pol, 2010; Watts & Strogatz, 1998), but to remain parsimonious, a clear conclusion is difficult, since the two factors are closely linked and with the present data, we cannot fully disentangle how these predictors link with learning relative to the motivation to learn (Fagiolo, 2007; van den Heuvel & Pol, 2010; Watts & Strogatz, 1998). The fact that the same characteristic that predicts effects of associative learning on perceived pain can also predict adherence to learning practices is an indicator that this system may play a central role in motivational aspects of learning. General personality factors such as compliance (Kripalani, Risser, Gatti, & Jacobson, 2009; Svarstad, Chewning, Sleath, & Claesson, 1999), suggestibility (Kotov, Bellman, & Watson, 2004), or openness to new experiences (Costa & McCrae, 1989, 1992) could potentially be related to such connectivity patterns.

The striatum plays an important role in learning and is linked with motivation (Balleine, Delgado, & Hikosaka, 2007). We have observed that local connectivity patterns of this region predict the effects of associative learning (Hashmi et al., 2014) and also adherence in this study. Learning requires an active process of participant engagement, and these findings underscore the fact that an underlying neurobiological capacity facilitates an individual’s capacity to persist during a training program. Many of the regions that were common in their association with adherence, regardless of the type of training prescribed in the two groups, were in subcortical areas. The globus pallidus and putamen showed a high degree centrality, and the accumbens showed stronger clustering coefficients in relation to adherence regardless of the type of practice. Since the striatum and basal ganglia play an important role in learning and motivation, it is speculated that higher connectivity within their local network neighborhood is a substrate for optimizing behaviors such as adherence that require motivation (Doyon et al., 1997; Voorn, Vanderschuren, Groenewegen, Robbins, & Pennartz, 2004). In contrast, nodes known for their role in meditation such as the default mode, anterior insula, and somatosensory cortices showed greater connectivity in individuals who were able to adhere to the meditation practice (Ives-Deliperi, Solms, & Meintjes, 2011). Furthermore, higher clustering in superior temporal gyrus and superior frontal gyrus predicted adherence to the practice of writing. These findings indicate that the capacity to adhere to a given mental exercise benefits from (a) optimized intrinsic connectivity in learning and motivational circuitry and (b) optimized intrinsic connectivity in regional substrates associated with the prescribed tasks, but these assumptions require more thorough investigation.

We now know that the idea that cognitive networks that mediate performance of mental tasks act in similar ways across individuals is a common misconception. Recent evidence suggests that these networks are not structured identically in every person (Tavor et al., 2016). Drawing inferences across groups can undermine the uniqueness and dispositional features that contribute to heterogeneity in how each person engages and responds to task demands. Functional connectivity profiles show systematic variations between individuals that appear to draw from past experiences along with developmental and genetic processes. Moreover, the way functional connections are optimized and segregated differs between individuals during brain development and across the life span (M. Y. Chan, Park, Savalia, Petersen, & Wig, 2014; Khan et al., 2018). Like “static” connectivity that represents the time-invariant aspects of the connectivity structure of the brain, brain networks also show dynamic and time-varying characteristics, and both are linked with behavior, albeit somewhat differently (Monti et al., 2014). While this study focused on establishing the link between network segregation in static networks and behavior, there are other studies that have measured flexibility of time-varying networks as a function of learning behaviors (Hastie et al., 2013, p. 72). Further research is needed to establish the mechanistic features of large-scale network dynamics that facilitate adherence.

The present observations are useful for understanding whether so-called functional networks observed with fMRI are important for mental functions. The upshot is that there is a link between baseline functional network topology and adherence to learning, and we may be able to use this information for predicting behavior. The large-scale topological properties that emerge on observing synchronous BOLD activity in spatially distributed regions may be a useful substrate for observing individual differences in the work space of cognitive decisions and functions (Kanai & Rees, 2011). Brain networks are self-organized to balance efficiency and cost by enhancing clustering and optimizing the number of short paths (E. T. Bullmore & Sporns, 2012). The functional significance of network topology remains unclear, but the alterations in this balance that occur during brain development (Khan et al., 2018; Power et al., 2010), in anesthesia (Boveroux et al., 2010; Hashmi et al., 2017), during task performance (Stevens et al., 2012), and in brain disorders (Yu et al., 2012) are evidence converging to suggest that topology has a role in mental function. Thus, the overlap between brain network topology and behavior is inconsistent between individuals of the same group and is a further indicator that we can improve precision and clinical utility of these findings by improving the resolution of brain behavior relationships through stratification and within-group classifications of data. Further studies are needed to precisely establish the categories of functions subserved by segregation measures and to elaborate what behavioral or neural functions are subserved or represented by network integration. Furthermore, by mapping domain-specific and domain-general properties to behavior, concise and useful information can be garnered to understand variations in human behavior such as motivation, cognitive abilities, and task performance.

To the best of our knowledge, this is the first study that uses brain data for a machine learning model to predict adherence to a trial. However, there is growing interest in applying machine learning to neuroimaging analysis to predict behavioral outcome (Doll, Jacobs, Sanfey, & Frank, 2009; Hoeft et al., 2011; Mansson et al., 2015). A common limitation across all neuroimaging studies is small sample size. For improving generalization ability caused by a small dataset, a cross-validation method can be used for validating the predictive performance of machine learning classifiers. Moreover, a large number of features result in a high amount of variance in machine learning models, which results in overfitting in scarce data. To prevent this, the backward elimination method was used (Horst & Macewan, 1960) to identify only a robust feature set.

After parameter optimization through LOOCV, the decision tree classifier achieved high accuracy in the binary classification problem using nodal measures of resting-state functional MRI as a classification feature. Moreover, comparing performance of random forest, AdaBoost, decision tree, and Naïve Bayes algorithms (Fernandez-Delgado, Cernadas, Barro, & Amorim, 2014) allowed us to test the predictive strength that was not reliant on one tool or by chance. Overall, the classifier’s performance establishes some useful parameters for predicting adherence with resting-state fMRI. We have shown that the classification approach is applicable for predicting adherence.

## CONCLUSIONS

Through this study, we have established that individuals vary in their ability to adhere to learning new skills and that the variability in brain connectivity may contribute to the ability to follow through on prescribed behavioral instructions. Thus, even before individuals undergo training, their brain connectivity patterns are predictive of their capacity to perform the prescribed exercises. This variability in adherence is associated with the extent of clustering and segregation of brain networks. In light of previous findings, these findings indicate the role of intrinsic patterns of the brain as a feature of the motivation to learn and, more specifically, as a conducive indicator of adherence to learning.

## SUPPORTING INFORMATION

Supporting information for this article is available at https://doi.org/10.1162/netn_a_00136.

## AUTHOR CONTRIBUTIONS

Marzie Saghayi: Conceptualization; Data curation; Formal analysis; Investigation; Methodology; Software; Validation; Visualization; Writing - Original Draft; Writing - Review & Editing. Jonathan Greenberg: Conceptualization; Data curation; Formal analysis; Resources; Writing - Review & Editing. Christopher O’Grady: Methodology; Software. Farshid Varno: Methodology; Resources; Software. Muhammad Ali Hashmi: Formal analysis; Investigation; Methodology; Project administration; Resources; Software; Supervision; Writing - Review & Editing. Bethany Bracken: Conceptualization; Funding acquisition; Investigation; Project administration; Resources; Writing - Review & Editing. Stan Matwin: Conceptualization; Investigation; Project administration; Resources; Software; Supervision; Validation; Writing - Review & Editing. Sara Lazar: Conceptualization; Data curation; Formal analysis; Funding acquisition; Investigation; Methodology; Project administration; Resources; Supervision; Validation; Writing - Review & Editing. Javeria Ali Hashmi: Conceptualization; Data curation; Formal analysis; Funding acquisition; Investigation; Methodology; Project administration; Resources; Software; Supervision; Validation; Writing - Original Draft; Writing - Review & Editing.

## FUNDING INFORMATION

Javeria Ali Hashmi, Natural Sciences and Engineering Research Council of Canada (http://dx.doi.org/10.13039/501100002790), Award ID: RGPIN/05684-2016. Sara Lazar, National Institutes of Health (http://dx.doi.org/10.13039/100000002), Award ID: AG048351. Sara Lazar, Intelligence Advanced Research Projects Activity (http://dx.doi.org/10.13039/100011039), Award ID: 2014-13121700006. Javeria Ali Hashmi, Canada Research Chairs (http://dx.doi.org/10.13039/501100001804), Award ID: 950-231109. Javeria Ali Hashmi, Canadian Foundation for Innovation (CFI), Award ID: 35702. Javeria Ali Hashmi, CIHR project grant, Award ID: 168878. Javeria Ali Hashmi, Nova Scotia Health Authority Research Fund.

## TECHNICAL TERMS

Adherence: How well an individual complies with instructions such as attending classes and follow-up on practicing the prescribed exercise.
Segregation in brain connectivity: Strength of within-network connectivity of brain regions compared with connectivity within the rest of the network.
Integration in brain connectivity: A global coordinative coupling of functionally distinct brain regions.
Hub: A node that has a disproportionately high number of node connections relative to other nodes.
Backward elimination: The process of iteratively removing irrelevant features. It begins with the full set of features, and in each iteration a feature is removed.
Generalization: A machine learning (ML)–related term that refers to how accurately a trained ML algorithm can predict data that were not used in training, or “unseen” data.
