# Supplementary material for: Brain network topology predicts participant adherence to mental training programs
Source: Netw Neurosci. 2020 Jul 1;4(3):528–55. doi: 10.1162/netn_a_00136 (PMC7462432; doi:10.1162/netn_a_00136)
Supplement: Supplementary file 1 [file netn-04-528-s001.pdf]

Saghayi, M., Greenberg, J., O'Grady, C., Varno, F., Hashmi,, M. A., Bracken, B., Matwin, S., Lazar, S. W., & Hashmi, J. A. (2020). Supporting information for "Brain network topology predicts participant adherence to mental training programs." *Network Neuroscience*, *4*(3), 528–555. https://doi.org/10.1162/netn_a_00136

Supplementary Table 1. Optimized Harvard-Oxford parcellation; 131 regions. Resting state networks, Anatomic brain regions, and associated Montreal Neurological Institute coordinates.

	**ROIs**		**MNI Coordinates**		
**N**	**Regions**	**Abbreviations**	**x**	**y**	**z**
1	Caudal anterior cingulate left	ACCc_L	-4	40	-2
2	Caudal anterior cingulate right	ACCc_R	4	40	-2
3	Mid anterior cingulate left	ACCm_L	-6	-2	42
4	Mid anterior cingulate right	ACCm_R	6	-2	42
5	Rostral anterior cingulate left	ACCr_L	-4	38	18
6	Rostral anterior cingulate mid posterior left	ACCcrm_L	-6	18	34
7	Rostral anterior cingulate mid posterior right	ACCcrm_R	6	18	34
8	Rostral anterior cingulate posterior left	ACCrp_L	-4	22	20
9	Rostral anterior cingulate posterior right	ACCrp_R	4	22	20
10	Rostral anterior cingulate right	ACCr_R	4	38	18
11	Subgenual anterior cingulate left	ACCsg_L	-4	16	-14
12	Subgenual anterior cingulate right	ACCsg_R	4	16	-14
13	Amygdala left	Amyg_L	-24	-4	-18
14	Amygdala right	Amyg_R	24	-4	-18
15	Angular gyrus left	Ang_L	-54	-56	26
16	Angular gyrus right	Ang_R	54	-56	26
17	Brain stem	BrStem	0	-26	-28
18	Caudate left	Caud_L	-12	14	8
19	Caudate right	Caud_R	12	14	8
20	Cingulate gyrus, posterior division left	Cingp_L	-4	-38	32
21	Cingulate gyrus, posterior division right	Cingp_R	4	-38	32
22	Central opercular cortex left	Cop_L	-48	-4	8
23	Central opercular cortex right	Cop_R	48	-4	8
24	Cuneal cortex left	Cun_L	-4	-82	30
25	Cuneal cortex right	Cun_R	4	-82	30
26	Dorsal anterior insula left	dINSa_L	-32	20	0
27	Dorsal anterior insula right	dINSa_R	32	20	0
28	Dorsal medial prefrontal cortex, anterior division left	dMPFCa_L	-4	50	28
29	Dorsal medial prefrontal cortex, anterior division right	dMPFCa_R	4	50	28
30	Dorsal medial prefrontal cortex, posterior division left	dMPFCp_L	-4	26	48
31	Dorsal medial prefrontal cortex, posterior division right	dMPFCp_R	4	26	48

32	Frontal orbital cortex left	FO_L	-40	30	-14
33	Frontal operculum cortex left	Fop_L	-40	20	4
34	Frontal operculum cortex right	Fop_R	40	20	4
35	Frontal orbital cortex right	FO_R	40	30	-14
36	Frontal pole left	FP_L	-30	54	20
37	Frontal pole right	FP_R	30	54	20
38	Globus pallidus left	GP_L	-16	-2	-2
39	Globus pallidus right	GP_R	16	-2	-2
40	Heschls gyrus (includes H1 and H2) left	He_L	-48	-18	6
41	Heschls gyrus (includes H1 and H2) right	He_R	48	-18	6
42	Hippocampus left	Hipp_L	-28	-22	-16
43	Hippocampus right	Hipp_R	28	-22	-16
44	Intracalcarine cortex left	IC_L	-6	-74	12
45	Intracalcarine cortex right	IC_R	6	-74	12
46	Inferior frontal gyrus, pars opercularis left	IFGpo_L	-54	14	16
47	Inferior frontal gyrus, pars opercularis right	IFGpo_R	54	14	16
48	Inferior frontal gyrus, pars triangularis left	IFGpt_L	-50	30	16
49	Inferior frontal gyrus, pars triangularis right	IFGpt_R	50	30	16
50	Middle insula left	INSm_L	-40	-2	-2
51	Middle insula right	INSm_R	40	-2	-2
52	Posterior insula left	INSp_L	-38	-14	8
53	Posterior insula right	INSp_R	38	-14	8
54	Inferior temporal gyrus, anterior division left	ITGa_L	-50	-6	-40
55	Inferior temporal gyrus, anterior division right	ITGa_R	50	-6	-40
56	Inferior temporal gyrus, posterior division left	ITGp_L	-56	-32	-24
57	Inferior temporal gyrus, posterior division right	ITGp_R	56	-32	-24
58	Inferior temporal gyrus, temporooccipital part left	ITGtp_L	-56	-54	-18
59	Inferior temporal gyrus, temporooccipital part right	ITGtp_R	56	-54	-18
60	Lingual gyrus left	Ling_L	-10	-68	-2
61	Lingual gyrus right	Ling_R	10	-68	-2
62	Lateral occipital cortex, inferior division left	LOcci_L	-48	-78	-2
63	Lateral occipital cortex, inferior division right	LOcci_R	48	-78	-2
64	Lateral occipital cortex, superior division left	LOccs_L	-40	-78	34
65	Lateral occipital cortex, superior division right	LOccs_R	40	-78	34
66	Middle frontal gyrus left	MFG_L	-40	20	44
67	Middle frontal gyrus right	MFG_R	40	20	44

68	Medial prefrontal cortex left	MPFC_L	-6	60	8
69	Medial prefrontal cortex right	MPFC_R	6	60	8
70	Middle temporal gyrus, anterior division left	MTGa_L	-58	-2	-22
71	Middle temporal gyrus, anterior division right	MTGa_R	58	-2	-22
72	Middle temporal gyrus, posterior division left	MTGp_L	-62	-22	-18
73	Middle temporal gyrus, posterior division right	MTGp_R	62	-22	-18
74	Middle temporal gyrus, temporooccipital part left	MTGto_L	-60	-52	0
75	Middle temporal gyrus, temporooccipital part right	MTGto_R	60	-52	0
76	Nucleus accumbens left	NAc_L	-10	10	-8
77	Nucleus accumbens right	NAc_R	10	10	-8
78	Occipital fusiform gyrus left	OccFG_L	-28	-76	-14
79	Occipital fusiform gyrus right	OccFG_R	28	-76	-14
80	Occipital pole left	OccP_L	-8	-100	6
81	Occipital pole right	OccP_R	8	-100	6
82	Orbito frontal pole left	OFP_L	-32	58	-6
83	Orbito frontal pole right	OFP_R	32	58	-6
84	Precuneous cortex left	pCun_L	-4	-64	38
85	Precuneous cortex right	pCun_R	4	-64	38
86	Parahippocampal gyrus, anterior division left	pHippa_L	-24	-6	-34
87	Parahippocampal gyrus, anterior division right	pHippa_R	24	-6	-34
88	Parahippocampal gyrus, posterior division left	pHippp_L	-24	-32	-18
89	Parahippocampal gyrus, posterior division right	pHippp_R	24	-32	-18
90	Planum polare left	PlP_L	-48	-4	-6
91	Planum polare right	PlP_R	48	-4	-6
92	Planum temporale left	PlT_L	-60	-22	8
93	Planum temporale right	PlT_R	60	-22	8
94	Parietal operculum cortex left	Pop_L	-48	-32	20
95	Parietal operculum cortex right	Pop_R	48	-32	20
96	Postcentral gyrus left	PostC_L	-54	-20	46
97	Postcentral gyrus right	PostC_R	54	-20	46
98	Precentral gyrus left	PreC_L	-44	-8	52
99	Precentral gyrus right	PreC_R	44	-8	52
100	Putamen left	Put_L	-30	-4	0
101	Putamen right	Put_R	30	-4	0
102	Supracalcarine cortex left	Sc_L	-2	-84	12
103	Supracalcarine cortex right	Sc_R	2	-84	12

104	Superior frontal gyrus left	SFG_L	-22	22	54
105	Superior frontal gyrus right	SFG_R	22	22	54
106	Supplementary motor area left	SMA_L	-4	-2	58
107	Supplementary motor area right	SMA_R	4	-2	58
108	Supra marginal gyrus left	SMGa_L	-58	-32	40
109	Supra marginal gyrus right	SMGa_R	58	-32	40
110	Supramarginal gyrus, posterior division left	SMGp_L	-60	-48	32
111	Supramarginal gyrus, posterior division right	SMGp_R	60	-48	32
112	Superior parietal lobule left	SPL_L	-32	-50	60
113	Superior parietal lobule right	SPL_R	32	-50	60
114	Superior temporal gyrus, anterior division left	STGa_L	-58	-4	-6
115	Superior temporal gyrus, anterior division right	STGa_R	58	-4	-6
116	Superior temporal gyrus, posterior division left	STGp_L	-66	-26	6
117	Superior temporal gyrus, posterior division right	STGp_R	66	-26	6
118	Temporal fusiform cortex, anterior division left	TFCa_L	-32	-6	-42
119	Temporal fusiform cortex, anterior division right	TFCa_R	32	-6	-42
120	Temporal fusiform cortex, posterior division left	TFCp_L	-36	-16	-32
121	Temporal fusiform cortex, posterior division right	TFCp_R	36	-16	-32
122	Thalamus left	Thal_L	-10	-18	8
123	Thalamus right	Thal_R	10	-18	8
124	Temporal occipital fusiform cortex left	TOF_L	-34	-54	-16
125	Temporal occipital fusiform cortex right	TOF_R	34	-54	-16
126	Temporal pole left	TP_L	-40	16	-30
127	Temporal pole right	TP_R	40	16	-30
128	Ventral anterior insula left	vINSa_L	-36	10	-14
129	Ventral anterior insula right	vINSa_R	36	10	-14
130	Ventral medial prefrontal cortex left	vMPFC_L	-4	50	-20
131	Ventral medial prefrontal cortex right	vMPFC_R	4	50	-20
